# Engineered *Escherichia coli* Nissle 1917 secreting anti-TNF-α nanobody as a single-strain live biotherapeutic for inflammatory bowel disease

**DOI:** 10.3389/fimmu.2026.1865862

**Published:** 2026-07-02

**Authors:** Qiumei Zhu, Shitao Feng, Zhende Yan, Zijian Wang, Xinran Huang, Changning Sun, Bingqiang Zhang, Xinrui Li, Yuchao Gu

**Affiliations:** 1 School of Medicine and Pharmacy, Ocean University of China, Qingdao, China; 2 Qingdao Center of Technology Innovation for Shark Antibody Development, College of Biological Engineering, Qingdao University of Science and Technology, Qingdao, China; 3 Qingdao Restore Medical Laboratory Co., Ltd., Qingdao, Shandong, China

**Keywords:** *Escherichia coli* Nissle 1917, gut microbiota, inflammatory bowel disease, live biotherapeutic, nanobody

## Abstract

**Background:**

The rising global incidence of inflammatory bowel disease (IBD) creates an urgent need for safer, gut-targeted therapies. Current treatments, from small-molecule drugs to systemic anti-tumor necrosis factor-alpha (TNF-α) biologics, are frequently limited by off-target immunosuppression, heightened infection risk, and poor mucosal bioavailability. Engineered probiotic-based live biotherapeutics offer a compelling alternative by enabling localized drug production within the inflamed intestine.

**Methods:**

We engineered *Escherichia coli* Nissle 1917 (EcN) to secrete the anti-TNF-α nanobody MT1, creating the streamlined, single-strain platform EcN-MT1. Five signal peptides were screened, and plasmid-based and CRISPR-Cas9-mediated chromosomal integration strategies were compared. Structural modeling and molecular dynamics simulated MT1−murine TNF-α (mTNF-α) binding. Binding affinity and anti-inflammatory activity were assessed by ELISA and in lipopolysaccharide (LPS)-stimulated RAW264.7 macrophages. Therapeutic efficacy was further evaluated in a dextran sulfate sodium (DSS)-induced murine colitis model by assessing body weight, disease activity index (DAI), colon length, histopathology, colonic pro-inflammatory cytokines, and 16S rRNA gut microbiota profiling.

**Results:**

Among the tested signal peptides, α-hemolysin (HlyA) achieved highest secretion (4.6 mg/L), and the plasmid-based strain markedly outperformed genomic integrants without impairing growth. Simulations confirmed stable complementarity-determining regions (CDR)-mediated binding, consistent with the high affinity (EC_50_ 27.9 nM) and potent suppression of LPS-induced mRNA expression of *Tnf* and interleukin-1β (*Il1b*) in macrophages. In the DSS-induced murine colitis model, oral administration of EcN-MT1 significantly attenuated weight loss, improved DAI scores, and preserved colon length. Histopathological analysis revealed reduced mucosal ulceration, crypt loss, and immune cell infiltration, accompanied by downregulated colonic *Tnf* and *Il1b* mRNA. Notably, EcN-MT1 treatment restored gut microbial diversity, corrected dysbiosis, and enriched beneficial taxa linked to butyrate production, barrier enhancement, and anti-inflammatory effects.

**Conclusion:**

This study establishes EcN-MT1 as a potent, orally deliverable live biotherapeutic that achieves localized TNF-α neutralization while concurrently promoting microbial and mucosal homeostasis, offering a novel and translatable strategy for IBD treatment.

## 1 Introduction

Inflammatory bowel disease (IBD), encompassing Crohn’s disease (CD) and ulcerative colitis (UC) (1–5), is a chronic, immune-mediated disorder driven by genetic susceptibility (6–9), environmental triggers (10), dysregulated immunity (6, 11), and gut microbiota alterations (7, 12). Despite advances in therapy, current treatments—including corticosteroids, immunomodulators, and systemically administered anti-tumor necrosis factor-alpha (anti-TNF-α) biologics—often fail to induce durable remission (13, 14) and carry significant risks such as systemic immunosuppression, opportunistic infections, and malignancies (15–19). These limitations highlight the urgent need for safer, gut-restricted therapeutic strategies that neutralize key inflammatory mediators without systemic exposure.

Live biotherapeutics based on engineered probiotics offer a promising solution by enabling localized drug production within the inflamed intestine. Among candidate chassis, *Escherichia coli* Nissle 1917 (EcN) stands out. It has been used clinically for over a century (e.g., in Mutaflor^®^) (20, 21), is endorsed by European Crohn’s and Colitis Organisation (ECCO) guidelines for UC maintenance therapy (22, 23), and exhibits superior gut-colonizing capacity (24) compared to transient colonizers like *Lactococcus lactis* (*L. lactis*) (25). While wild-type EcN provides modest anti-inflammatory benefits, its therapeutic potency is insufficient to target central drivers such as TNF-α, a master cytokine that amplifies inflammation via immune cell activation, pro-inflammatory signaling, and barrier disruption (26–29).

Advances in synthetic biology now enable engineering of EcN to secrete therapeutic proteins (30–33). This opens the door to combining EcN’s aforementioned advantages with antibody-based TNF-α neutralization for potent, localized effects. However, achieving clinically relevant payload levels remains challenging due to EcN’s limited native secretion capacity—a limitation that is especially pronounced for conventional antibodies, whose large size and multi-chain complexity impede efficient bacterial secretion. Nanobodies, a novel class of antibody fragments derived from the variable domains of camelid heavy-chain-only antibodies (34), offer an ideal solution. Unlike conventional antibodies, they lack light chains and constant domains, which simplifies genetic engineering. As the smallest naturally occurring antigen-binding fragments (~15 kDa), they possess excellent tissue penetration (35), enabling them to reach the intestinal submucosa. They also resist low pH, heat, and proteolytic degradation, retaining biological activity during gastrointestinal transit (36). Importantly, nanobodies exhibit target affinity and specificity comparable to those of conventional antibodies, yet can be produced cost-effectively in microbial expression systems without the need for complex refolding steps (37). Collectively, these properties make nanobodies uniquely suited for oral delivery via engineered probiotics.

Among nanobodies targeting TNF-α, MT1 is particularly well-characterized. Originally isolated from a llama immunized with mTNF-α (38), MT1 has been engineered into various formats and successfully expressed in diverse hosts (38–40). The TNF-α-neutralizing activity of MT1 and its variants has been validated in multiple proof-of-concept studies, including intestinal delivery via *L. lactis* (38) or complex EcN platforms (41, 42). However, these existing systems either employ *L. lactis*, a transient colonizer inherently less suited for sustained gut delivery than EcN, or rely on dual-strain or multi-gene circuits that introduce genetic and manufacturing complexity, hindering clinical translation.

To address this, we developed a streamlined, single-strain live biotherapeutic platform, designated EcN-MT1. It consists of EcN carrying a single plasmid that directs high-level secretion of MT1, via an optimized secretion system. We hypothesized that oral administration of EcN-MT1 would achieve potent, localized TNF-α neutralization in the colon, thereby ameliorating inflammation while avoiding systemic toxicity. Here, we demonstrate that EcN-MT1 significantly alleviates disease severity in a dextran sulfate sodium (DSS)-induced murine colitis model, suppresses mucosal pro-inflammatory cytokines, preserves epithelial architecture, and restores gut microbial homeostasis. Our findings establish EcN-MT1 as a potent, simplified, and translatable platform for next-generation IBD therapy.

## 2 Materials and methods

### 2.1 Materials and reagents

The probiotic strain EcN was obtained from Biobw. The pEcCas9 and pEcgRNA plasmids were provided by the Institute of Microbiology, Chinese Academy of Sciences. pBAD24 plasmids containing different signal peptide genes were maintained in the laboratory. The cell line RAW264.7 was obtained from the Stem Cell Bank, Chinese Academy of Sciences.

### 2.2 Preparation of competent cells

EcN was streaked onto LB agar plates and a single colony was inoculated into 5 mL of LB broth. The overnight culture was diluted and grown to logarithmic growth phase. Cells were harvested by centrifugation at 4, 000 × g for 10 min at 4 °C. The pellet was washed three times with ice-cold transformation buffer (10 mM 1, 4-Piperazinediethanesulfonicacid (PIPES) (dissolved in 1 M KOH), 55 mM MnCl_2_, 15 mM CaCl_2_, 250 mM KCl). Finally, cells were resuspended in transformation buffer containing 10% (v/v) dimethyl sulfoxide (DMSO), aliquoted, flash-frozen in liquid nitrogen, and stored at −80 °C.

### 2.3 Preparation of EcN-Cas9 electrocompetent cells

The pEcCas9 plasmid was transformed into chemically competent EcN cells via heat shock. A single positive colony was inoculated into 5 mL of LB medium supplemented with antibiotic and cultured overnight at 37 °C with shaking at 180 rpm. The overnight culture was diluted 1:100 into fresh LB medium containing 10 mM L−arabinose (Macklin, # L800478) to induce Cas9 protein expression, and incubated until the optical density at 600 nm (OD_600_) reached approximately 0.6. Cells were harvested by centrifugation at 4, 000 × g for 10 min at 4 °C. The pellet was washed three times with sterile, ice−cold 10% (v/v) glycerol. After the final wash, the cell pellet was gently resuspended in a small volume of 10% glycerol, aliquoted, flash-frozen in liquid nitrogen, and stored at −80 °C.

### 2.4 Genome knock-in

To achieve stable genomic integration of the MT1 expression cassette, CRISPR-Cas9 technology was employed (43, 44). The *amyA* locus was selected as the insertion site. Primers containing the N20 targeting sequence and homologous arms to the pEcgRNA backbone were designed. Using these primers, the pEcgRNA plasmid was amplified by PCR to generate a linearized vector. The template plasmid was then digested with *Dpn* I (TAKARA, #1609). The linearized product was subjected to homologous recombination for recircularization. The resulting mixture was transformed into DH5α competent cells, and positive clones were verified by Sanger sequencing (Tsingke Biotechnology Co., Ltd., Beijing, China). A correct clone was selected for plasmid amplification and extraction, yielding the pEcgRNA-*amyA* vector. The donor DNA fragment, comprising the MT1 expression cassette driven by selected promoters and flanked by homologous arms targeting the *amyA* locus, was assembled via overlap extension PCR. The purified donor fragment and the pEcgRNA-*amyA* plasmid were co-electroporated into EcN-Cas9 electrocompetent cells. After recovery, transformants were selected on appropriate antibiotic plates. Positive clones were initially screened by colony PCR. Correct integration and sequence integrity were further confirmed by Sanger sequencing. Finally, plasmid elimination was performed to obtain marker-free engineered strains.

### 2.5 Western blot analysis

Protein samples were separated by 15% sodium dodecyl sulfate-polyacrylamide gel electrophoresis (SDS-PAGE) and transferred onto polyvinylidene difluoride (PVDF) membranes (Merck Millipore, #IPVH00005). Membranes were blocked with 5% (w/v) skim milk in Tris-buffered saline containing 0.1% Tween-20 (TBST) at room temperature for 1 h. Incubation with primary antibody (mouse anti-6×His tag, Proteintech, #66005-1-Ig, 1:2000) was performed overnight at 4 °C. After washing, membranes were incubated with horseradish peroxidase (HRP)-conjugated secondary antibody (goat anti-mouse IgG, Merck Millipore, #AP124P, 1:10, 000) for 1 h. Protein bands were visualized using an enhanced chemiluminescence (ECL) detection kit (Epizyme Biotech, #PG112). The protein amounts were quantified using Image J software, relative to the loading control.

### 2.6 Protein expression and purification

The gene encoding MT1 was cloned into pBAD24 vectors and transformed into EcN. A single colony was inoculated into LB medium containing ampicillin (100 μg/mL) and grown overnight at 37 °C. The culture was diluted 1:100 into fresh medium and grown to logarithmic growth phase. Protein expression was induced with 0.5% (w/v) L-arabinose for 6 h. Cells were harvested by centrifugation. For each gram of wet cell pellet, 10 mL of TES buffer (30 mM Tris-HCl, 1 mM EDTA, 20% sucrose, pH 8.0) supplemented with protease inhibitor cocktail (TargetMol, #C0001) was added. The suspension was incubated on ice with vortexing for 30 min, followed by centrifugation. The pellet was then resuspended in 10 mL of ice-cold MgSO_4_ solution (30 mM Tris-HCl, 5 mM MgSO_4_, pH 8.0) per gram and incubated on ice for another 30 min with agitation. The supernatant containing periplasmic proteins was collected, filtered, and subjected to purification using Ni-NTA affinity beads (Smart-Lifesciences, #SA005100), following the manufacturer’s instructions. Protein purity was assessed by SDS-PAGE.

### 2.7 Monitoring of bacterial growth curves

Single colonies of each strain were inoculated into 5 mL of LB broth and cultured overnight at 37 °C with shaking at 180 rpm. The overnight cultures were then diluted 1:100 into fresh LB medium. Bacterial growth was monitored by measuring the optical density at 600 nm (OD_600_) at 2-hour intervals over a period of 10 h, following a previously described procedure (45). Growth curves were generated by plotting OD_600_ against time.

### 2.8 Enzyme-linked immunosorbent assay

ELISA was used to assess MT1 affinity as previously described (46). 96-well plates were coated with 2 μg/mL recombinant mTNF-α (mTNF-α; Novoprotein, #CF09) in phosphate-buffered saline (PBS) overnight at 4 °C. After blocking with PBS containing 5% (w/v) skim milk, serial dilutions of purified MT1 were added. Plates were incubated with HRP-conjugated anti-6×His tag antibody (AlpVHHs, # 004-101-005, 1:10, 000). Color development used 3, 3′, 5, 5′-tetramethylbenzidine (TMB; Solarbio, #PR1200), stopped with 1 M H_2_SO_4_. Absorbance was measured at 450 nm (OD_450_).

### 2.9 Cell culture and anti-inflammatory assay in RAW264.7 macrophages

RAW264.7 cells were cultured in Dulbecco’s Modified Eagle Medium (DMEM; Gibco, #11965092) supplemented with 10% fetal bovine serum (FBS; PAN Biotech, #ST30−3302) and 1% penicillin−streptomycin at 37 °C, 5% CO_2_. Cells were seeded in 6-well plates at 2 × 10^5^ cells per well. After pretreatment with or without MT1 (1 μg/mL) for 2 h, inflammation was induced with 100 ng/mL lipopolysaccharide (LPS) for 24 h. Total RNA was extracted using SparkZol Reagent (Sparkjade, #AC0101). cDNA was synthesized using the 1st Strand cDNA Synthesis Kit (Yeasen, #11141ES60). Quantitative real−time PCR (qPCR) was performed using SYBR Green master mix (Sparkjade, #AH0101) according to the manufacturer’s protocol with minor modifications, as previously described (47).

### 2.10 Molecular docking

The three-dimensional structure of mTNF−α was retrieved from the Protein Data Bank (PDB ID: 7kp7). The structural model of the nanobody MT1 was predicted using AlphaFold3 (https://alphafoldserver.com/). Molecular docking was performed with the HDOCK server (http://hdock.phys.hust.edu.cn/), treating mTNF−α as a homotrimeric complex. The complementarity-determining region (CDR) of MT1 were defined as the putative binding site. The top−ranking complex model with the lowest binding energy was selected and saved in PDB format. Protein-ligand interaction patterns were visualized using PyMOL, and hydrogen−bond interactions were extracted for analysis.

### 2.11 Molecular dynamics simulation

The docked MT1-mTNF−α complex was prepared using the CHARMM−GUI web server. The system was protonated at pH 7.0 and parameterized with the CHARMM36 force field. All simulations were performed with GROMACS 2023.3. After energy minimization and equilibration in the NPT ensemble, a 200 ns molecular dynamics (MD) simulation was performed at 310 K with a 2 fs time step. Trajectories were saved every 10 ps. The stability and dynamics of the complex were evaluated by calculating the root−mean−square deviation (RMSD), root−mean−square fluctuation (RMSF), radius of gyration (Rg), solvent−accessible surface area (SASA), and free−energy landscape (FEL). To confirm the stability of the simulation, a representative conformation from the most stable simulation segment was extracted and examined for any dissociation or major conformational changes.

### 2.12 Animal experiment

All animal experimental protocols were reviewed and approved by the Animal Ethics Committee of Qingdao University of Science and Technology (Approval No. QKDLL-2025-169). Twenty male C57BL/6J mice (6–8 weeks old, 18–22 g) were purchased from Shandong Pengyue Laboratory Animal Technology Co., Ltd. and housed in a climate−controlled environment (24 ± 1 °C) with a 12 h light/dark cycle. After one week of acclimation, the mice were randomly assigned to four experimental groups (n = 5 per group): (1) Water + PBS (control group), (2) DSS + PBS (model group), (3) DSS + EcN, and (4) DSS + EcN−MT1. Mice were group-housed by experimental group to avoid cross-group microbiota exchange. Throughout the entire experiment, all mice received drinking water containing 0.5% L−arabinose. From day 1 to day 7, the DSS + EcN and DSS + EcN−MT1 groups received a daily oral gavage of 1 × 10^9^ colony-forming units (CFU) suspended in 120 μL PBS, whereas the control and model groups received an equal volume of PBS alone. Colitis was induced from day 2 to day 6 by providing drinking water containing 3% (w/v) dextran sodium sulfate (DSS; ApexBio Technology, #9011-18-1, molecular weight 35-45 kDa) to all groups except the control. Body weight, stool consistency, and fecal bleeding were monitored and scored daily. The disease activity index (DAI) was calculated based on an established scoring system (48), which combines scores for weight loss, stool consistency, and fecal bleeding. The detailed scoring criteria are provided in **Supplementary Table S3**. Humane endpoints were predefined as >20% body weight loss combined with additional clinical signs, including inability to ambulate or access food and water, hypothermia, or labored respiration; animals meeting these criteria would be euthanized immediately. No mice reached these endpoints before the scheduled termination of the experiment. On day 12, mice were euthanized by cervical dislocation. Colons were excised, flushed with ice−cold sterile saline, measured for length, and then snap−frozen in liquid nitrogen for subsequent analysis.

### 2.13 Histopathological staining

Colon tissues were flushed with ice−cold saline to remove luminal contents and immediately fixed in 4% paraformaldehyde solution (Sparkjade, #EE0001). After fixation, tissues were dehydrated through a graded ethanol series, cleared in xylene, embedded in paraffin, and sectioned at 4 μm thickness. Sections were deparaffinized, rehydrated, and stained with hematoxylin and eosin (H&E) for microscopic examination. Histopathological changes were evaluated and compared across groups.

### 2.14 RNA extraction and qPCR from colon tissues

Frozen colon tissues (30–100 mg) were homogenized in SparkZol Reagent. Total RNA extraction, cDNA synthesis, and qPCR were performed as described in Section 2.9.

### 2.15 16S rRNA gene sequencing

Fresh fecal samples were collected at the endpoint of the experiment, immediately snap-frozen in liquid nitrogen, and stored at −80 °C until analysis. Total bacterial genomic DNA was extracted from feces. The V3-V4 region of the bacterial 16S rRNA gene was amplified using the universal primers 338F and 806R. PCR products were subjected to high-throughput sequencing analysis on the Illumina MiSeq platform (Majorbio Bio-Pharm Technology Co., Ltd., Shanghai, China). Microbial α−diversity within samples was assessed using the Chao1 richness estimator, Ace index, observed species (Sobs), Shannon diversity index, and Simpson index. β−diversity analysis, based on Bray-Curtis distances, was performed to evaluate compositional differences between groups, visualized via non-metric multidimensional scaling (NMDS). In the NMDS plot, 95% confidence ellipses were drawn around group centroids to aid visualization of group separation. Linear discriminant analysis effect size (LEfSe) was used to identify differentially abundant bacterial taxa among groups. In this analysis, statistical significance was first assessed using the non-parametric Kruskal-Wallis (KW) sum-rank test to identify taxa with differential abundance across all groups, followed by the Wilcoxon rank-sum test for pairwise comparisons between groups. Taxa with a *P* < 0.05 in these statistical tests were retained. Subsequently, the effect size of each differentially abundant taxon was estimated using linear discriminant analysis (LDA). Among the statistically significant taxa, those with a logarithmic LDA score > 2.0 were considered biologically relevant.

### 2.16 Statistical analysis

Data are presented as mean ± standard error of the mean (SEM). Statistical comparisons between two groups were performed using unpaired two-tailed Student’s *t*-test. *P < 0.05, **P < 0.01, ***P < 0.001.

## 3 Results

### 3.1 Optimization of anti-TNF-α nanobody secretion in EcN

**Figure 1:**
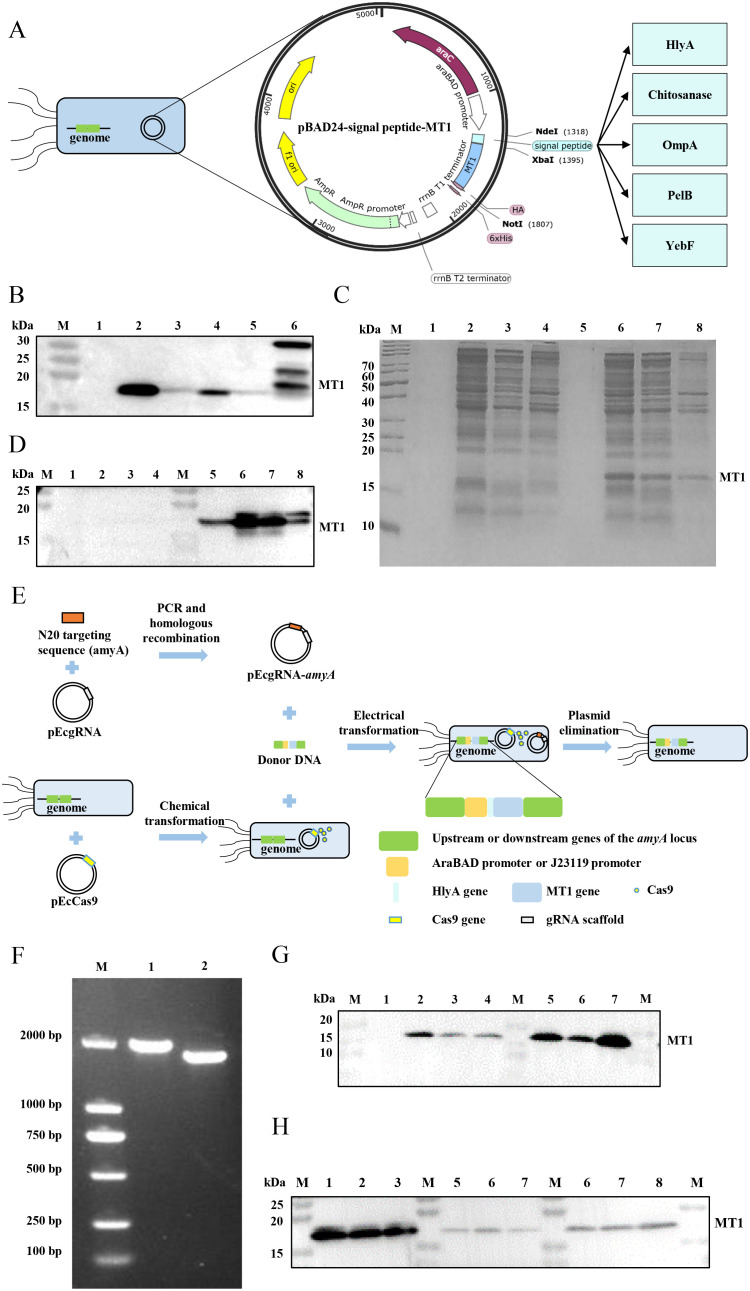
Optimization of anti-TNF-α nanobody secretion in EcN. **(A)** Schematic representation of pBAD24-based plasmid constructs encoding the MT1 nanobody fused to one of five signal peptides: α-hemolysin (HlyA), chitosanase, outer membrane protein A (OmpA), PelB, or YebF. **(B)** Western blot analysis of MT1 in culture supernatants. M, prestained protein marker; Lane 1: wild-type EcN. Lanes 2–6: EcN strains expressing MT1 fused to the HlyA, chitosanase, OmpA, PelB, and YebF signal peptides, respectively. **(C)** SDS-PAGE of protein fractions from wild-type EcN (lanes 1–4) and the HlyA-MT1 strain (lanes 5–8): M, prestained protein marker; lane 1/5, culture supernatant (secreted protein fraction obtained by centrifugation of the culture); lane 2/6, total lysate after sonication (intracellular fraction obtained by sonication of the pelleted cells); lane 3/7, soluble fraction (supernatant post-centrifugation); lane 4/8, insoluble fraction (pellet). **(D)** Western blot of the samples shown in **(C)**, probed with anti-His antibody. **(E)** Strategy for CRISPR-Cas9-mediated genomic integration of the MT1 expression cassette. **(F)** Agarose gel electrophoresis of colony PCR products confirming successful integration of the two donor DNA fragments. M, DNA marker; Lane 1: donor 1 (upstream arm-arabinose promoter-HlyA-MT1-downstream arm). Lane 2: donor 2 (upstream arm-J23119 promoter-HlyA-MT1-downstream arm). **(G)** Western blot analysis of MT1 secretion from genomically integrated strains. M, prestained protein marker; Lane 1: wild-type EcN; lanes 2–4: three biological replicates of the strain with the arabinose-inducible araBAD promoter; lanes 5–7: three biological replicates with the constitutive J23119 promoter. **(H)** Comparison of MT1 secretion levels among strains by Western blot. Lane M, prestained protein marker. Lanes 1–3: three biological replicates of the plasmid-based EcN-MT1 strain. Lanes 4–6: three biological replicates of the genomically integrated strain with the arabinose-inducible araBAD promoter. Lanes 7–9: three biological replicates of the genomically integrated strain with the constitutive J23119 promoter. Data are representative of three independent experiments.

The therapeutic efficacy of engineered live biotherapeutics depends critically on achieving sufficient local concentrations of functional protein payloads. A key limitation of EcN as a delivery chassis is its inherently low capacity for secreting heterologous proteins into the extracellular environment. To overcome this, we evaluated five N-terminal signal peptides—α-hemolysin (HlyA), chitosanase, outer membrane protein A (OmpA), PelB, and YebF—for their ability to direct secretion of the anti-TNF-α nanobody MT1. Each signal peptide was fused in-frame to the MT1 coding sequence and cloned into a pBAD24 expression vector under the control of an arabinose-inducible promoter (**Figure 1A**). Following transformation into EcN and induction with arabinose, all cultures were normalized to the same OD_600_ prior to sample collection to ensure equal cell numbers were compared across strains. Culture supernatants and cell lysates were analyzed by Western blot. Among the candidates, HlyA mediated the highest level of extracellular MT1 with minimal intracellular retention, establishing it as the optimal secretion signal (**Figures 1B–D**).

Next, we compared two genetic strategies for stable MT1 expression: a plasmid-based system versus chromosomal integration. Using CRISPR-Cas9 technology, we integrated the HlyA-MT1 cassette into the EcN chromosome (**Figure 1E**), generating two variants driven by either the inducible araBAD promoter or the constitutive synthetic promoter J23119 (**Figure 1F**; **Supplementary Figure 1**). After plasmid elimination, both integrants secreted detectable MT1 (**Figure 1G**). However, quantitative Western blot analysis revealed that the secretion titers from both genomically integrated strains were substantially lower than those achieved by the plasmid-bearing strain (**Figure 1H**).

Given that high local concentration of the nanobody is essential for effective TNF-α neutralization in the inflamed colon, we selected the plasmid-based strain, designated EcN-MT1, for all subsequent *in vitro* and *in vivo* studies. This decision prioritizes therapeutic potency in the current proof-of-concept phase.

### 3.2 Functional characterization of EcN-MT1 strain and MT1 nanobody

**Figure 2:**
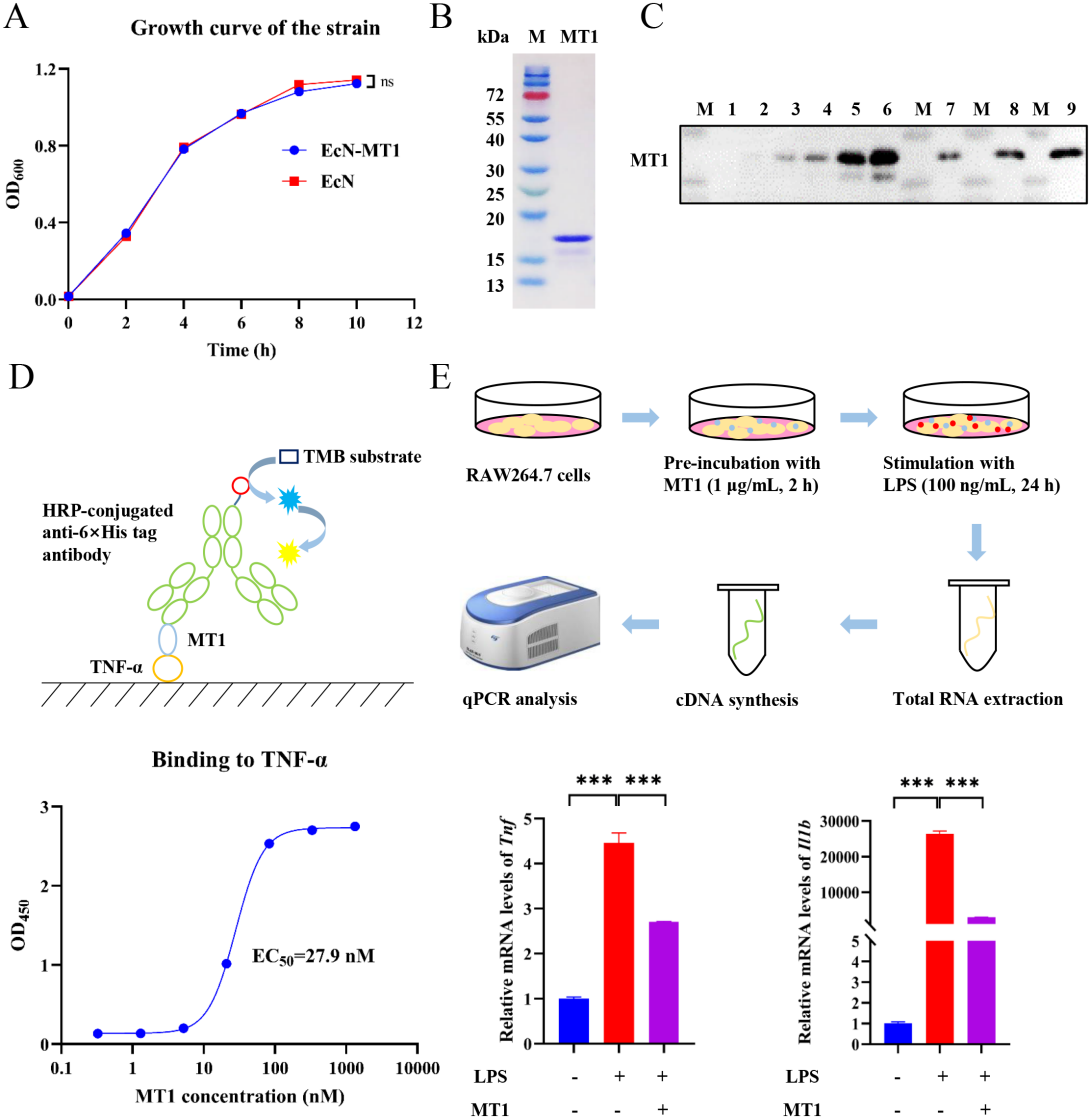
Functional characterization of EcN-MT1 strain and MT1 nanobody. **(A)** Growth curves of wild-type EcN and EcN-MT1 in LB medium at 37 °C, monitored by optical density at 600 nm (OD_600_). **(B)** SDS-PAGE of purified MT1 protein isolated from EcN-MT1 culture supernatant via immobilized metal-affinity chromatography (IMAC). M, prestained protein marker. **(C)** Quantification of MT1 secretion by Western blot densitometry using purified MT1 as a standard. M, prestained protein marker; Lanes 1–6: two-fold serial dilutions of MT1 standard (0–45 ng); lanes 7–9: four-fold dilution, two-fold dilution, and undiluted (neat) culture supernatant from EcN-MT1, respectively. **(D)** ELISA showing dose-dependent binding of purified MT1 to mTNF-α. Top: schematic of the indirect ELISA. mTNF-α (orange hollow circles) is coated on the plate; His-tagged MT1 (blue hollow circles) binds to mTNF-α; HRP-conjugated anti-6×His antibody (HRP shown as orange solid circle) binds to the His-tagged MT1. HRP catalyzes TMB oxidation, producing a blue color that turns yellow after acid stop, and the absorbance is read at 450 nm. Bottom: binding curve of MT1 to mTNF-α, with a half maximal effective concentration (EC_50_) of 27.9 nM. **(E)** Anti-inflammatory effect of MT1 in LPS-stimulated RAW264.7 macrophages. Cells were pre-treated with 1 µg/mL MT1 for 2 h, then stimulated with 100 ng/mL LPS for 24 h. mRNA levels of *Tnf* (left) and *Il1b* (right) were measured by quantitative real-time PCR (qPCR), normalized to Actb. Data are mean ± SEM of n = 3 independent experiments. Statistical significance was assessed by two-tailed Student’s *t*-test: ***P < 0.001, ns, not significant.

To assess potential fitness costs associated with plasmid carriage, we compared the growth kinetics of wild-type EcN and EcN-MT1 in LB medium. Both strains exhibited nearly identical growth curves (**Figure 2A**), indicating that harboring the MT1 expression plasmid does not impair basic physiology of EcN. The C-terminal 6×His tag enabled efficient purification of recombinant MT1 via immobilized metal-affinity chromatography (IMAC) (**Figure 2B**). Quantification by densitometric analysis of Western blot, calibrated against a purified MT1 standard, revealed a secretion titer of 4.6 mg/L in the culture medium (**Figure 2C**; **Supplementary Figure 3**)—a level sufficient to support functional studies.

We next evaluated the binding affinity of MT1 for mTNF-α by ELISA. Serial dilutions of MT1 produced a dose-dependent signal on mTNF-α–coated plates, with a half maximal effective concentration (EC_50_) of 27.9 nM (**Figure 2D**). This nanomolar-range EC_50_ indicates strong functional binding and is consistent with effective target engagement in physiological settings.

To confirm anti-inflammatory activity, we tested purified MT1 in LPS-stimulated RAW264.7 macrophages. As expected, LPS challenge induced robust upregulation of pro-inflammatory cytokine mRNAs, including *Tnf* and *Il1b*. Strikingly, pre-treatment with purified MT1 significantly suppressed the LPS-induced expression of both cytokines at the transcriptional level (**Figure 2E**), demonstrating its potent immunomodulatory function *in vitro*.

### 3.3 Computational modeling of the MT1–mTNF-α interaction

**Figure 3:**
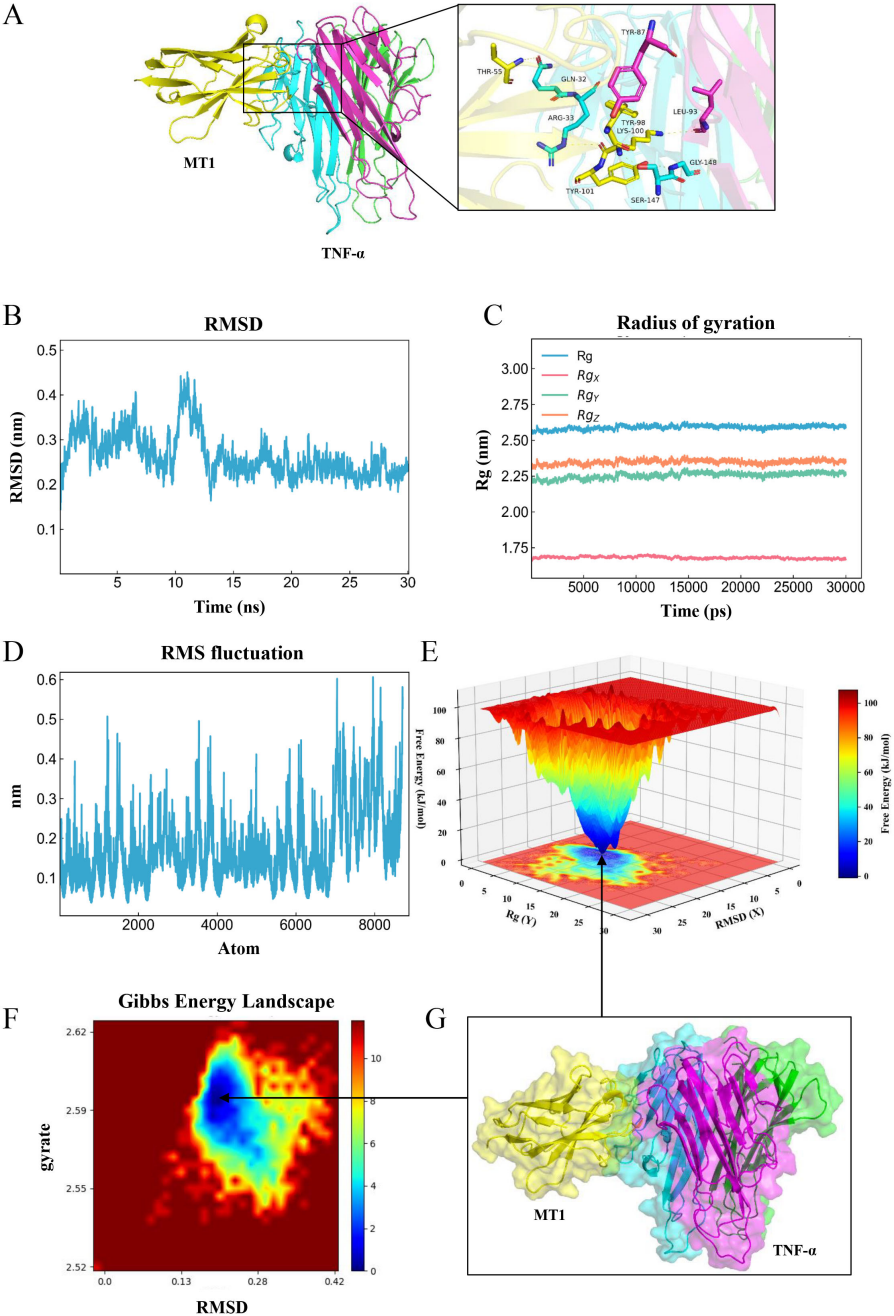
Computational modeling of the MT1–mTNF-α interaction. **(A)** Docked structural model of the MT1-mTNF-α complex. Interfacial residues are shown as sticks, and hydrogen bonds are indicated by yellow dashed lines. **(B)** Backbone root−mean−square deviation (RMSD) of the complex during molecular dynamics (MD) simulation. **(C)**The radius of gyration (Rg) of the complex over time, reflecting overall compactness. **(D)** Per-residue root−mean−square fluctuation (RMSF) of the complex during MD simulation. **(E)** Three-dimensional free-energy landscape (FEL) of the complex. The landscape is projected onto the RMSDs of the complex along three principal axes (X, Y, and Z). Energy values are given in kJ/mol, with the lowest energy basin highlighted in blue. **(F)** Two-dimensional FEL projected onto RMSD (x-axis) and gyrate (y-axis). The lowest-energy basin is highlighted in blue. **(G)** Representative structure (most stable conformation) of the MT1–mTNF-α complex extracted from the global free-energy minimum identified in **(E, F)**.

Although the functional neutralization of mTNF-α by MT1 has been established (38, 39), the detailed three-dimensional structure of the MT1-mTNF-α complex has not been experimentally resolved. Prior studies have not reported the specific binding interface or key interacting residues. Therefore, as a first step toward understanding the molecular mechanism of their interaction and to provide a structural rationale for the high-affinity binding observed in our ELISA (EC_50_ = 27.9 nM), we performed computational docking and MD simulations. We note that these predictions provide a structural hypothesis that remains to be experimentally validated. The docking model predicted a stable binding interface primarily mediated by the CDR of MT1 (**Figure 3A**). Specifically, the interaction network is dominated by hydrogen bonds involving residues from CDR1 and CDR3. Thr55 in CDR1 forms a hydrogen bond with Gln32 of mTNF-α, while CDR3 contributes multiple hydrogen bonds via Tyr98 (to Tyr87), Lys100 (to Leu93, Arg33, and Ser147) and Tyr101 (to Gly148). The figure highlights these key hydrogen−bonding interactions, hydrophobic contacts and salt bridges are not shown.

The stability of the docked complex was validated by MD simulations. The RMSD of the complex reached a plateau after ~30 ns, indicating equilibration (**Figure 3B**). The Rg remained stable throughout the simulation (**Figure 3C**), confirming overall structural compactness. Residue-level flexibility analysis revealed elevated RMSF (RMSF > 0.4 nm) primarily in loop regions of both MT1 and mTNF-α, suggesting these flexible loops may participate in conformational adjustments during binding (**Figure 3D**). Moreover, the SASA of the complex remained stable within a narrow range (245-265 nm^2^) after the initial 5 ns, further supporting the structural integrity of the complex (**Supplementary Figure 4**).

To identify the most stable binding conformation, we calculated the FEL using RMSD and Rg as reaction coordinates (**Figures 3E, F**). The global minimum (deep blue region) on the FEL corresponds to a low-energy, high-population conformational cluster. The representative structure extracted from this minimum reveals that MT1 stably docks into a groove of the mTNF-α trimer (**Figure 3G**). Collectively, these computational results suggest a structural rationale for the high-affinity interaction between MT1 and mTNF-α, highlighting the predicted critical role of the CDR loops in stabilizing the complex. Experimental validation of these predictions, for example through site-directed mutagenesis of the identified key residues, would be required to confirm their functional importance.

### 3.4 Oral administration of EcN-MT1 ameliorates acute DSS-induced colitis in mice

**Figure 4:**
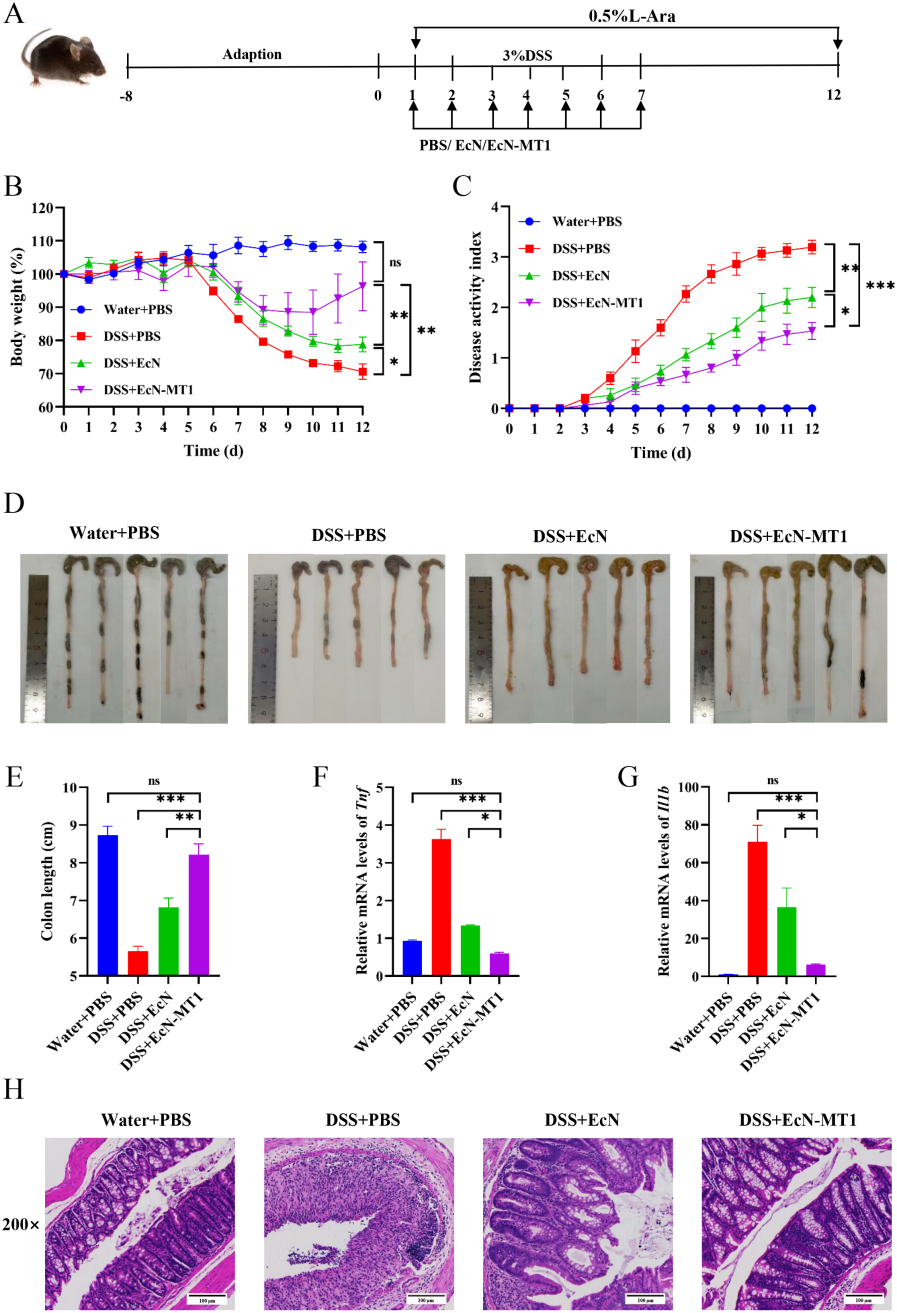
Oral administration of EcN-MT1 ameliorates acute DSS-induced colitis in mice. **(A)** Schematic illustration of the experimental timeline and treatment regimen. **(B, C)** Assessment of disease severity through **(B)** daily body weight change (expressed as percentage of initial weight) and **(C)** the composite disease activity index (DAI). **(D)** Representative photograph and **(E)** quantitative analysis of colon lengths from each treatment group. **(F, G)** Quantitative real-time PCR (qPCR) analysis of pro-inflammatory cytokine mRNA levels in colon tissues: **(F)** *Tnf*, and **(G)** *Il1b*. **(H)** Representative H&E-stained histological sections of colon tissues. Data are presented as mean ± SEM. Statistical significance was determined by unpaired two-tailed Student’s *t*-test for comparisons between groups. *P < 0.05, **P < 0.01, ***P < 0.001, ns, not significant.

To evaluate the therapeutic efficacy of EcN-MT1 *in vivo*, we employed a murine model of acute colitis induced by DSS. As shown in **Figure 4A**, mice received daily oral gavage of either PBS (vehicle), wild-type EcN or EcN-MT1 (1 × 10^9^ CFU in 120 µL PBS) from day 1 to day 7, while colitis was induced by 3% DSS in drinking water from days 1-4, followed by regular water thereafter. Disease progression was monitored daily by assessing body weight, stool consistency, and presence of occult/gross blood—parameters used to calculate the DAI. As expected, DSS-treated mice receiving PBS exhibited progressive weight loss that persisted through day 7 (**Figure 4B**; **Supplementary Figure 5**). In contrast, EcN-MT1 administration significantly attenuated weight loss compared to both PBS and wild-type EcN groups. Consistently, DAI scores were highest in the PBS group, modestly reduced with EcN, and most effectively suppressed by EcN-MT1 (**Figure 4C**; **Supplementary Figure 6**).

To confirm intestinal delivery of the therapeutic payload, fecal samples were analyzed by Western blot. MT1 protein was detectable in the feces of EcN-MT1-treated mice (**Supplementary Figure 7**), consistent with secretion of the nanobody into the gut lumen. At study endpoint, colon length, a key indicator of inflammation severity, was significantly shortened in the PBS group, partially preserved with EcN, and fully maintained in the EcN-MT1 group, comparable to healthy controls (**Figures 4D, E**). To exclude the possibility that the superior therapeutic effect of EcN-MT1 was driven by a colonization advantage, we quantified the bacterial loads of wild-type EcN and EcN-MT1 in colonic tissues by qPCR using EcN-specific primers. The two strains showed comparable colonization levels, with no statistically significant difference (**Supplementary Figure 8**). This result indicates that the enhanced protection conferred by EcN-MT1 is attributable to MT1 secretion rather than to differential colonization. Consistently, qPCR analysis of colonic tissue revealed that DSS challenge markedly upregulated *Tnf* and *Il1b* mRNA levels. While EcN treatment partially suppressed these cytokines, EcN-MT1 induced significantly greater downregulation (**Figures 4F, G**). Colons from PBS-treated mice showed extensive ulceration, crypt destruction, and dense immune infiltration. EcN afforded moderate protection, whereas EcN-MT1 preserved crypt architecture, maintained epithelial integrity, and markedly reduced inflammatory cell influx, resulting in a histological appearance that closely resembled that of healthy tissue (**Figure 4H**).

Collectively, these results demonstrate that oral delivery of EcN-MT1 effectively alleviates acute DSS-induced colitis through localized TNF-α neutralization, leading to improved clinical outcomes, preserved colon length, reduced mucosal inflammation, and near-complete histological recovery.

### 3.5 EcN-MT1 restores gut microbial homeostasis in DSS-induced colitis mice

Gut microbiota dysbiosis is a hallmark of IBD (49). To investigate whether the therapeutic effects of EcN-MT1 is associated with modulation of the intestinal microbiome, we performed 16S rRNA gene sequencing on fecal samples collected at the endpoint of the colitis experiment.

**Figure 5:**
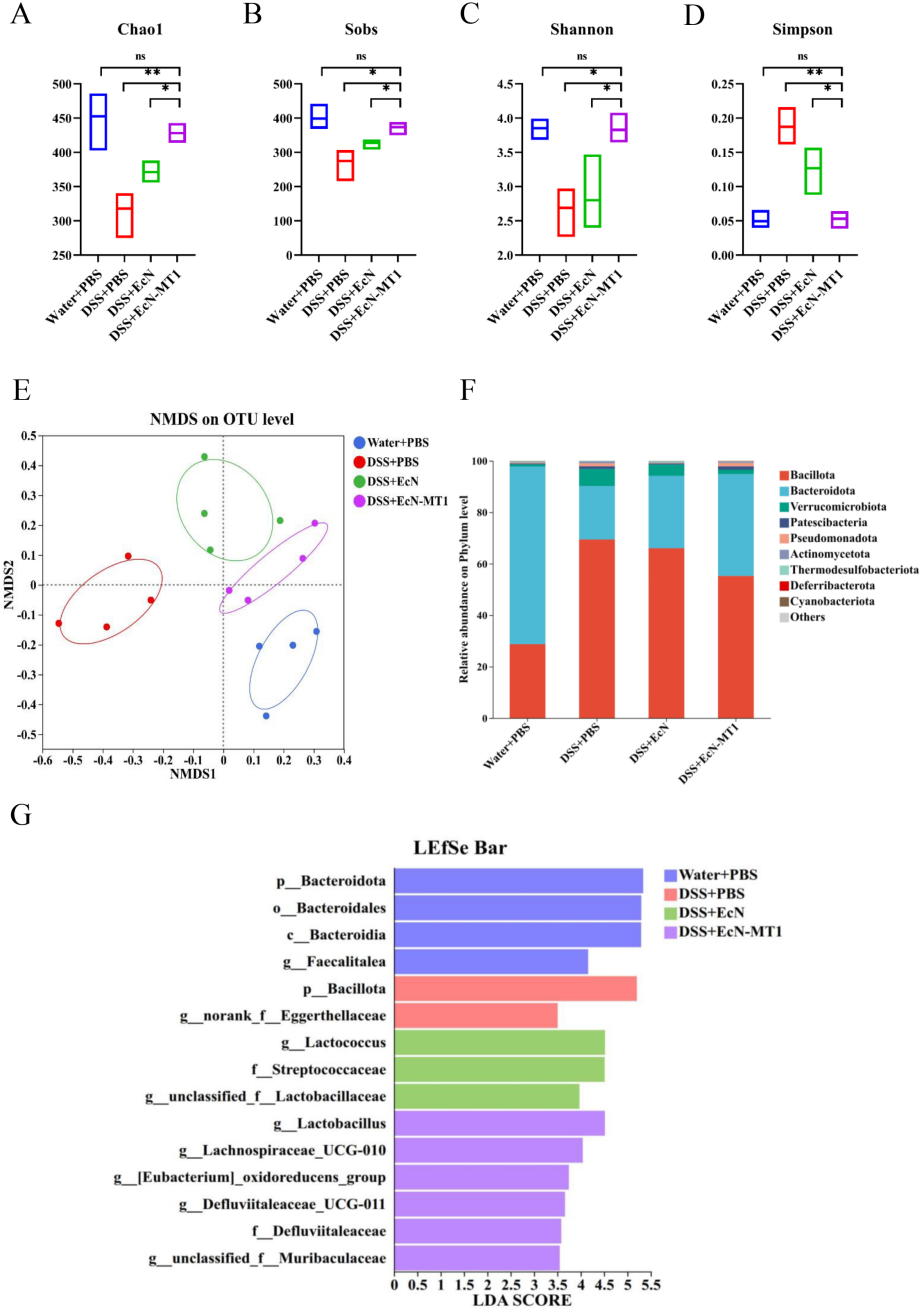
EcN-MT1 restores gut microbial homeostasis in DSS-induced colitis mice. **(A–D)** α-diversity indices (Chao1, Sobs, Shannon, Simpson) of the gut microbiota across different groups. **(E)** NMDS plot based on Bray-Curtis distances showing β-diversity among groups (n = 4 per group). Ellipses represent 95% confidence regions around the group centroids. One DSS + PBS sample was excluded due to insufficient fecal material. **(F)** Relative abundance of the top 10 bacterial phyla in the gut microbiota of each group. **(G)** Linear discriminant analysis effect size (LEfSe) bar plot displaying bacterial taxa (from phylum to genus level) that were statistically significant (*P* < 0.05, Kruskal-Wallis and Wilcoxon tests) and biologically relevant (linear discriminant analysis (LDA) score ≥ 2) across groups. Bar length corresponds to the LDA score, and labeled taxa are signature microbial taxa specific to their respective groups. Data are presented as mean ± SEM. Statistical significance was determined by unpaired two-tailed Student’s *t*-test for comparisons between groups. *P < 0.05, **P < 0.01, ns, not significant.

We first assessed α-diversity, using Chao1 and Sobs indices for microbial richness, and Shannon and Simpson indices for overall diversity (higher Shannon and lower Simpson values indicate greater diversity). DSS-treated mice exhibited significantly reduced microbial richness (lower Chao1 and Sobs indices) and overall diversity (reduced Shannon and elevated Simpson indices) compared to healthy controls (**Figures 5A–D**). Ace richness estimates showed highly concordant results and are presented in **Supplementary Figure 9**. Oral administration of EcN-MT1 markedly reversed these changes, restoring α-diversity to levels comparable to those of the control group. In contrast, wild-type EcN treatment conferred only a partial recovery. To examine compositional differences between groups (β-diversity), we performed NMDS based on Bray-Curtis distances. The microbial communities from DSS-treated mice clustered distinctly from those of healthy controls (**Figure 5E**). Both EcN and EcN-MT1 treatments shifted the community structure toward the control cluster, with the EcN-MT1 group showing the closest proximity, indicating more effective restoration of microbial composition.

At the phylum level, DSS-induced colitis mice were associated with profound dysbiosis (**Figure 5F**). Healthy controls were dominated by *Bacteroidota* (69.0%) and *Bacillota* (28.7%). DSS treatment drastically reduced *Bacteroidota* to 20.8% while increasing *Bacillota* to 69.4%, resulting in a markedly decreased *Bacteroidota*/*Bacillota* ratio—a signature commonly observed in IBD patients (50–52). Concurrently, *Verrucomicrobiota* expanded, consistent with its association with intestinal inflammation (53). EcN-MT1 induced a more pronounced restoration of the *Bacteroidota*/*Bacillota* balance, increasing *Bacteroidota* to 39.6% and reducing *Bacillota* to 55.3%, compared to 28.2% and 66.0% with wild-type EcN, respectively. Notably, only EcN-MT1 significantly suppressed the DSS-driven expansion of *Verrucomicrobiota*.

LEfSe identified bacterial taxa differentially enriched across groups (**Figure 5G**). The control group was characterized by *Bacteroidota*, including the health-associated genus *Faecalitalea* (54). In contrast, the DSS group showed enrichment of *Bacillota* and an unclassified genus from the family *Eggerthellaceae*. Of note, a member of this family, *Eggerthella*, has been reported to be more abundant in inflammatory bowel diseases and other inflammatory conditions (55). Wild-type EcN increased the abundance of *Lactococcus* (known for antimicrobial activity) (56), and members of *Streptococcaceae*. Strikingly, EcN-MT1 uniquely enriched a broader spectrum of protective taxa (57–64), all of which have been associated with intestinal barrier enhancement, butyrate production, or anti-inflammatory effects: *Lactobacillus*, *Lachnospiraceae _UCG-010*, *Muribaculaceae*, *[Eubacterium]_oxidoreducens_group*, *Defluviitaleaceae* and its genus *Defluviitaleaceae_UCG-011*. This multifaceted enrichment profile underscores the superior capacity of EcN-MT1 in restoring a homeostatic microbial ecology.

## 4 Discussion

In this study, we engineered EcN to secrete MT1, a high-affinity anti-TNF-α nanobody, and demonstrated that oral administration of EcN-MT1 significantly alleviated DSS-induced colitis in mice. Treatment with EcN-MT1 attenuated body weight loss, reduced DAI scores, preserved colon length, improved histopathological architecture, lowered pro-inflammatory cytokine levels, and restored gut microbial diversity and composition. Collectively, these findings establish EcN-MT1 as a promising orally delivered live biotherapeutic platform for localized TNF-α neutralization in intestinal inflammation.

Compared to prior probiotic-based delivery systems, such as the *L. lactis* strain secreting anti-TNF-α nanobodies reported by Vandenbroucke et al. (39), our EcN-MT1 platform offers several potential advantages. First, EcN is a human-origin probiotic with a well-established safety profile and superior gut-colonizing capacity, which is crucial for sustained local drug delivery (20–23). qPCR analysis confirmed that EcN-MT1 remained detectable in colonic tissues at the study endpoint at levels comparable to wild-type EcN (**Supplementary Figure 8**), indicating effective intestinal persistence throughout the treatment period. Second, EcN-MT1 achieved a high nanobody secretion titer of 4.6 mg/L in culture, which is approximately four times higher than that reported for *L. lactis* (1.08 mg/L) (39).This enhanced secretion capacity, combined with the plasmid retention rate of approximately 60% after 66 generations without antibiotic selection (**Supplementary Table S2**) and stable secretion over 60 generations (**Supplementary Figure 2**), likely enables more effective local TNF-α neutralization, which may underlie the enhanced therapeutic outcomes observed in our DSS model. Third, EcN is highly amenable to genetic engineering, facilitating integration of advanced synthetic biology modules—such as inducible promoters, sensing circuits, and targeting modalities—for precise spatiotemporal control of drug release, thereby improving both efficacy and safety profiles (65). Notably, while many existing EcN platforms rely on multi-target strategies or complex secretion machinery, our work demonstrates that a streamlined, single-strain, single-target approach, can achieve significant therapeutic benefit. Additionally, the use of L-arabinose as an inducer may confer ancillary benefits, as it has been reported to exert anti-inflammatory effects in murine colitis through modulation of the gut microbiota (66). However, this potential synergy was not directly evaluated in the current study.

The therapeutic efficacy of EcN-MT1 likely arises from two complementary mechanisms: (1) localized neutralization of TNF-α at the inflamed mucosal interface, leading to dampened NF-κB/MAPK signaling and reduced innate immune activation; and (2) probiotic-mediated support of mucosal homeostasis, including restoration of microbial balance and enhancement of epithelial barrier integrity. These effects are consistent with the observed improvements in dysbiosis and tissue histology. Specifically, 16S rRNA gene sequencing revealed that EcN-MT1 treatment effected a comprehensive restoration of the gut microbial ecology. Beyond rescuing α-diversity loss and correcting key dysbiotic features—including the *Bacteroidota*/*Bacillota* imbalance and *Verrucomicrobiota* expansion—EcN-MT1 selectively enriched functionally distinct beneficial taxa. Notably, it promoted butyrate-producing bacteria (*Lachnospiraceae_UCG-010* (57–59) and *[Eubacterium]_oxidoreducens_group*) (60); butyrate is known to strengthen epithelial tight junctions, modulate immune responses, and exert anti-inflammatory effects (60). Concurrently, EcN-MT1 enriched anti-inflammatory-associated taxa (*Defluviitaleaceae* and *Defluviitaleaceae_UCG-011*) (61, 62) as well as barrier-enhancing *Lactobacillus* (63) and health-associated *Muribaculaceae* (57, 64), collectively fostering a multifaceted protective microbial consortium. Thus, the enhanced therapeutic efficacy of EcN-MT1 is closely linked to its dual action: directly neutralizing TNF-α while simultaneously reshaping the dysbiotic microbiota toward a homeostatic state, creating a favorable ecological niche that synergizes to alleviate intestinal inflammation.

Nevertheless, several limitations should be acknowledged. First, although we normalized cultures by OD_600_ to ensure equal cell numbers across strains in our secretion comparison, we did not include a cytoplasmic protein marker (e.g., GroEL or DnaK) as an internal loading control, and thus cannot fully account for potential differences in metabolic output among strains. Second, the current plasmid-based system, although chosen for its higher secretion levels compared with chromosomal integration, relies on an antibiotic resistance marker and is therefore not ideal for clinical translation. In addition, the anti-inflammatory activity of MT1 was validated using purified protein; direct comparison with EcN-MT1 culture supernatant remains to be performed to assess efficacy in its native context. Furthermore, our 16S rRNA gene sequencing analysis was performed on a single animal experiment, and the mice were group-housed by experimental group. Although group-housing prevents cross-group microbiota exchange, it can introduce a co-housing effect via coprophagy that may homogenize the microbiota within each cage and amplify treatment-associated differences. Moreover, the DSS-induced colitis model primarily reflects epithelial damage and innate immune activation and does not fully recapitulate the adaptive immune involvement or transmural inflammation characteristic of human IBD, particularly Crohn’s disease. Finally, while we assessed phenotypic, histological, and cytokine endpoints, we did not systematically dissect the causal links between bacterial colonization, immune cell dynamics (e.g., Treg/Th17 balance, macrophage polarization), and mucosal repair.

Looking forward, several strategies could address these limitations and enhance the translational potential of EcN-MT1. First, future secretion studies should incorporate a cytoplasmic protein marker as an internal loading control to enable more rigorous strain-to-strain comparisons. Second, the development of antibiotic-free, genomically integrated expression platforms or self-regulating genetic circuits could overcome current limitations of plasmid based delivery. Additionally, a head-to-head comparison of purified MT1 versus EcN-MT1 culture supernatant would clarify whether other secreted factors modulate MT1 efficacy. Furthermore, independent replication of the microbiome findings in additional animal cohorts—ideally with individually housed or litter-matched designs—would help disentangle treatment effects from co-housing artifacts and strengthen the generalizability of our conclusions. Moreover, validation in additional IBD relevant models—such as interleukin-10 (IL-10) knockout mice, trinitrobenzene sulfonic acid (TNBS) induced colitis, or humanized intestinal systems—would strengthen the preclinical relevance of our findings. Finally, integrative multi-omics approaches—including single cell RNA sequencing, flow cytometry, and metabolomics—could elucidate the detailed immunomodulatory and barrier restorative mechanisms underlying EcN-MT1 efficacy.

## 5 Conclusion

In conclusion, this study demonstrates that oral delivery of an engineered EcN strain secreting an anti-TNF-α nanobody represents a viable strategy for targeted therapy in intestinal inflammation. By synergizing the colonization fitness and safety of EcN with the stability and potency of nanobodies, this platform offers a novel path toward safer, more localized IBD treatments.

## Data Availability

The datasets presented in this study can be found in online repositories. The names of the repository/repositories and accession number(s) can be found below: CNP0009430 (https://db.cngb.org/data_resources/project/CNP0009430/).

## Glossary

IBD: Inflammatory bowel disease
TNF-α: Tumor necrosis factor-alpha
EcN: *Escherichia coli* Nissle 1917
LPS: Lipopolysaccharide
DSS: Dextran sulfate sodium
DAI: Disease activity index
IL-1β: Interleukin-1β
CD: Crohn’s disease
UC: Ulcerative colitis
ECCO: European Crohn’s and Colitis Organisation
*L. lactis*: *Lactococcus lactis*
PIPES: 1, 4-Piperazinediethanesulfonicacid
DMSO: dimethyl sulfoxide
OD_600_: optical density at 600 nm
SDS-PAGE: Sodium dodecyl sulfate-polyacrylamide gel electrophoresis
PVDF: Polyvinylidene difluoride
TBST: Tris-buffered saline containing 0.1% Tween-20
HRP: Horseradish peroxidase
ECL: Enhanced chemiluminescence
ELISA: Enzyme-linked immunosorbent assay
PBS: Phosphate-buffered saline
TMB: 3, 3′, 5, 5′-tetramethylbenzidine
OD_450_: optical density at 450 nm
DMEM: Dulbecco’s Modified Eagle Medium
FBS: Fetal bovine serum
qPCR: Quantitative real-time PCR
CDR: Complementarity-determining region
MD: Molecular dynamics
RMSD: Root-mean-square deviation
RMSF: Root-mean-square fluctuation
Rg: Radius of gyration
SASA: Solvent-accessible surface area
FEL: Free-energy landscape
CFU: colony-forming units
H&E: Hematoxylin and eosin
NMDS: Non-metric multidimensional scaling
LEfSe: Linear discriminant analysis effect size
LDA: Linear discriminant analysis
HlyA: α-hemolysin
OmpA: outer membrane protein A
IMAC: Immobilized metal-affinity chromatography
EC_50_: Half maximal effective concentration
IL-10: interleukin-10
TNBS: trinitrobenzene sulfonic acid.
