# Supplementary material for: Engineered Escherichia coli Nissle 1917 secreting anti-TNF-α nanobody as a single-strain live biotherapeutic for inflammatory bowel disease
Source: Front Immunol. 2026 Jul 2;17:1865862. doi: 10.3389/fimmu.2026.1865862 (PMC13372642; doi:10.3389/fimmu.2026.1865862)
Supplement: Supplementary file 1 [file Supplementaryfile1.docx]

Supplementary Materials

1. **Supplementary Tables**

**Table S1. Amino acid sequences of nanobody MT1**

|  | Amino acid sequences |
| --- | --- |
| MT1 | GSQVQLQDSGGGLVQAGGSLRLSCAASGGTFSSIIMAWFRQAPGKEREFVGAVSWSGGTTVYADSVLGRFEISRDSARKSVYLQMNSLKPEDTAVYYCAARPYQKYNWASASYNVWGQGTQVTVSS |

**Table S2. Plasmid retention rate of EcN‑MT1 during serial passage without antibiotic selection.**

| Passage number | Cumulative Generations | Plasmid retention rate (%) |
| --- | --- | --- |
| 0 | 0 | 100 |
| 1 | 7 | 100 |
| 2 | 13 | 100 |
| 3 | 20 | 100 |
| 4 | 27 | 100 |
| 5 | 33 | 88.5 |
| 6 | 40 | 83.5 |
| 7 | 46 | 82 |
| 8 | 53 | 77.5 |
| 9 | 60 | 66 |
| 10 | 66 | 60.4 |

The strain was passaged every 12 h at 1:100 dilution. Generations per passage ≈ 6.64 (rounded). Retention rate = (Amp⁺ CFU / Amp⁻ CFU) × 100%.

**Table S3. Disease Activity Index (DAI) scoring parameters.**

| Score | Weight loss (%) | Stool consistency | Fecal blood |
| --- | --- | --- | --- |
| 0 | None | Normal, formed | None |
| 1 | 1–5 | Slightly loose | Small presence |
| 2 | 5–10 | Loose | Significant presence |
| 3 | 10–15 | Diarrhea | Gross blood |
| 4 | >15 |  |  |

DAI = (Weight Loss Score + Stool Consistency Score + Fecal Blood Score) / 3

1. **Supplementary Figures**

**
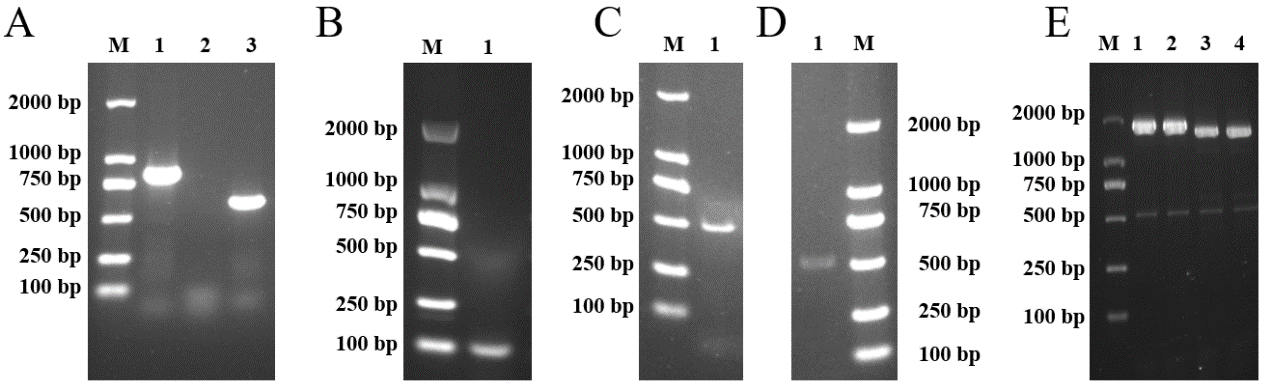
**

**Supplementary Figure 1. Agarose gel electrophoresis analysis of PCR-amplified donor fragments for genome integration.**
(A) PCR amplification of individual regulatory and coding elements: Lane 1, araBAD promoter-HlyA-MT1 fragment; Lane 2, J23119 promoter; Lane 3, HlyA-MT1 coding fragment.
(B) Confirmation of the J23119 promoter amplicon.
(C) Amplification of the 500 bp upstream homologous arm flanking the genomic insertion site.
(D) Amplification of the 500 bp downstream homologous arm flanking the genomic insertion site.
(E) Assembly PCR of the final donor constructs: Lanes 1-2, donor 1 (upstream arm-arabinose promoter-HlyA-MT1-downstream arm); Lanes 3-4, donor 2 (upstream arm-J23119 promoter-HlyA-MT1-downstream arm).


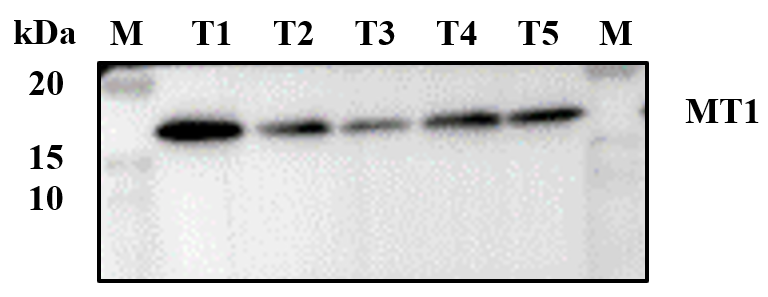


Supplementary Figure 2. Long‑term secretion stability of EcN‑MT1.

The strain was continuously passaged in antibiotic‑free LB medium for 5 days (approximately 60 generations). At each day (T1–T5), an aliquot was subjected to arabinose induction under standard conditions. MT1 secretion levels in the culture supernatants were analyzed by Western blot using an anti‑His antibody.


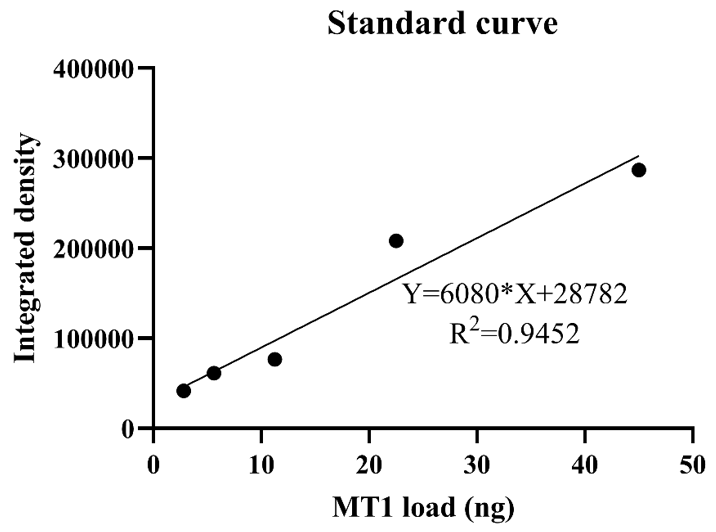


**Supplementary Figure 3. Standard curve for quantitative analysis of secreted MT1.**

A standard curve was generated by plotting the band intensity (determined by ImageJ) against known concentrations of purified MT1.


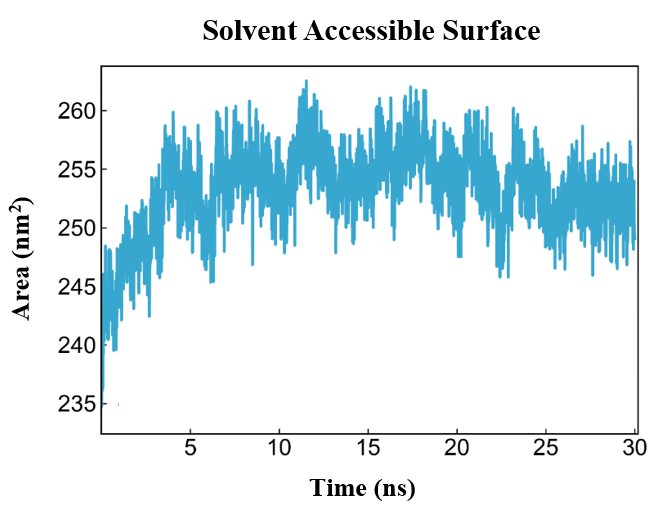


**Supplementary Figure 4. The solvent‑accessible surface area (SASA) of the MT1–mTNF-α complex.**


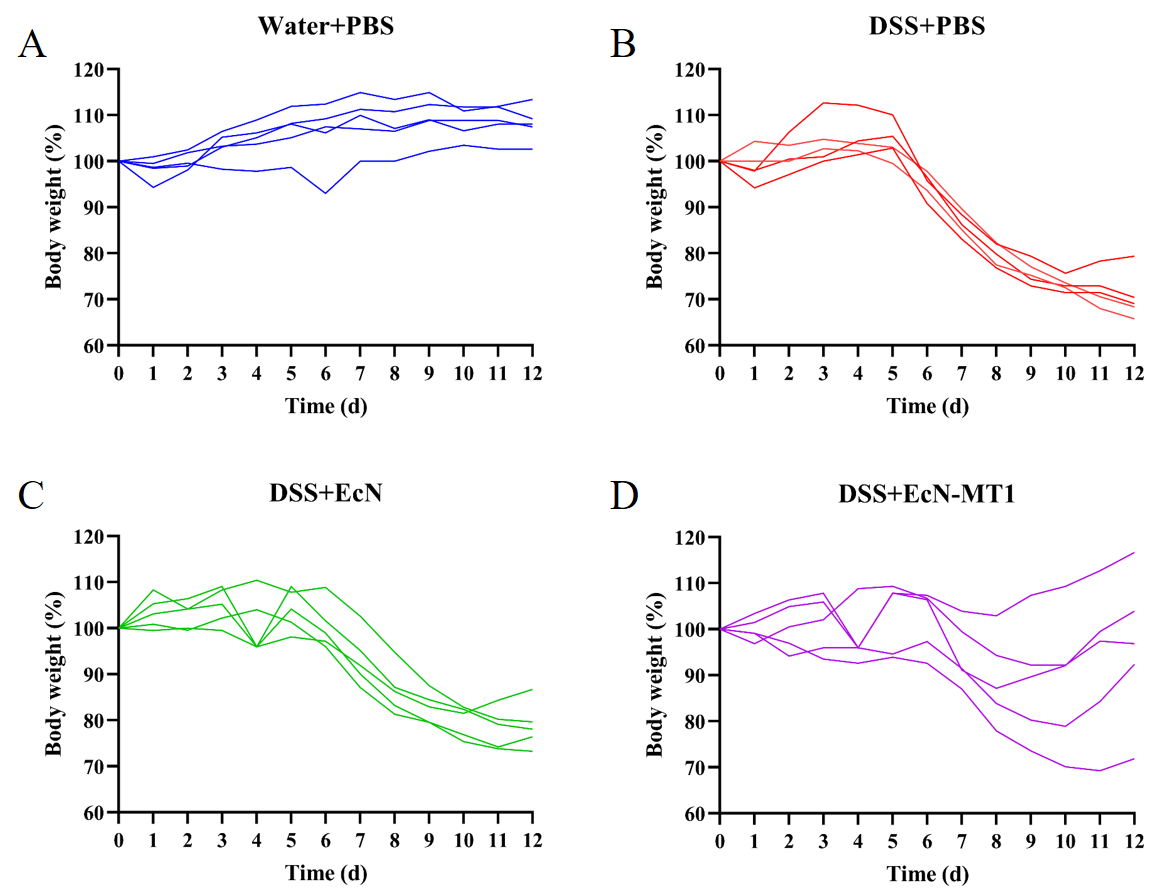


**Supplementary Figure 5. Daily body weight changes of individual mice in each group.**Body weight was monitored daily and expressed as percentage of initial weight.

(A) Water + PBS (control) group.

(B) DSS + PBS (model) group.

(C) DSS + EcN group.

(D) DSS + EcN-MT1 group.

Curves represent data from individual mice (n = 5 per group).


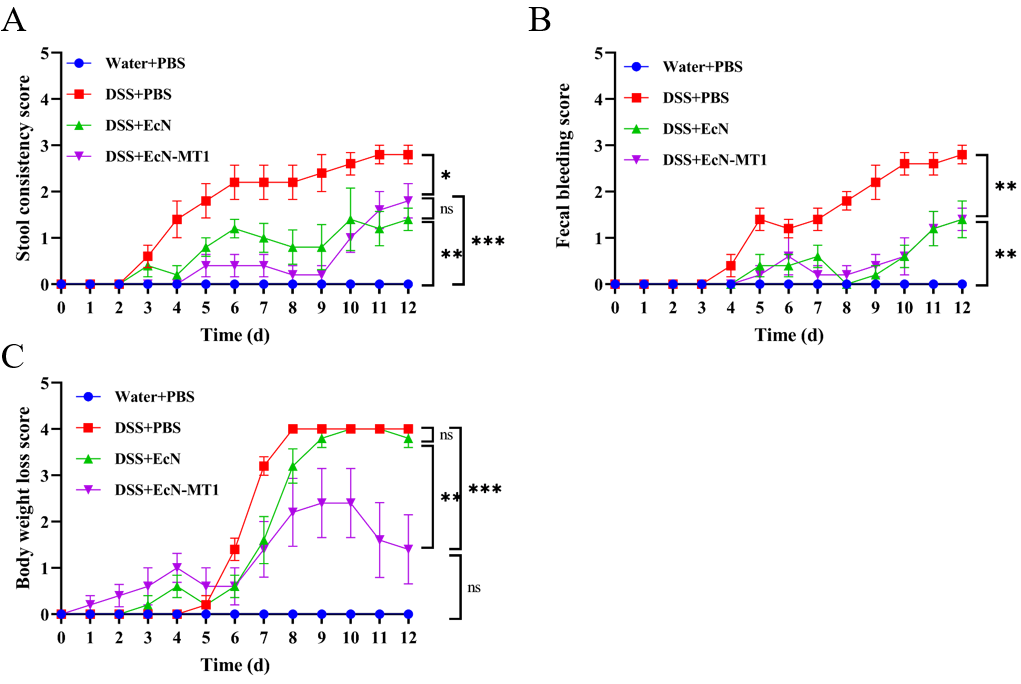


**Supplementary Figure 6. Individual DAI subscores during the course of DSS-induced colitis.**

(A) Body weight loss score.

(B) Stool consistency score.

(C) Fecal bleeding score.

Data are presented as mean ± SEM. Statistical significance was determined by unpaired two-tailed Student's *t*-test for comparisons between groups. **P* < 0.05, ***P* < 0.01, ****P* < 0.001, ns, not significant.


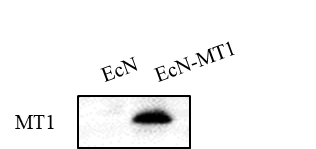


**Supplementary Figure 7. Western blot detection of MT1 protein in fecal samples.**

**
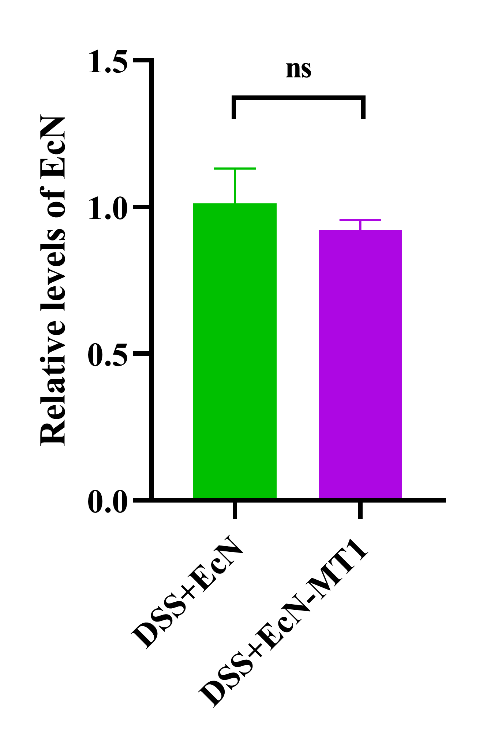
**

**Supplementary Figure 8. Quantification of EcN colonization in colonic tissues by qPCR.**

Total RNA was extracted from colonic tissues of DSS+EcN and DSS+EcN‑MT1 groups at the study endpoint, reverse‑transcribed into cDNA, and analyzed by qPCR using EcN‑specific primers. EcN levels were normalized to mouse *Gapdh* and are presented as relative levels.

Data are presented as mean ± SEM. Statistical significance was determined by unpaired two-tailed Student's *t*-test for comparisons between groups. ns, not significant.

**
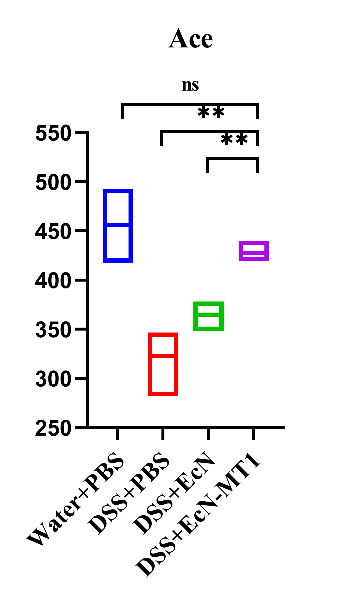
**

**Supplementary Figure 9.** Ace richness estimates of the gut microbiota across different groups.

Data are presented as mean ± SEM. Statistical significance was determined by unpaired two-tailed Student's *t*-test for comparisons between groups. **P* < 0.05, ***P* < 0.01, ****P* < 0.001, ns, not significant.
